# Bactericidal activity of avian complement: a contribution to understand avian-host tropism of Lyme borreliae

**DOI:** 10.1186/s13071-021-04959-0

**Published:** 2021-09-06

**Authors:** Valerie Sürth, Isabel Lopes de Carvalho, Maria Sofia Núncio, Ana Cláudia Norte, Peter Kraiczy

**Affiliations:** 1 Institute of Medical Microbiology and Infection Control, University Hospital, Goethe University Frankfurt, Frankfurt, Germany; 2 Centre for Vectors and Infectious Diseases Dr. Francisco Cambournac, National Institute of Health Doutor Ricardo Jorge, Lisbon, Portugal; 3 Institute of Environmental Health (ISAMB), Lisbon, Portugal; 4 Department of Life Sciences, Faculty of Sciences and Technology, MARE-Marine and Environmental Sciences Centre, University of Coimbra, Calçada Martim de Freitas, 3000-456 Coimbra, Portugal

**Keywords:** Spirochetes, *Borrelia*, Tick, *Ixodes*, Innate immunity, Immune evasion, Complement, Avian, Birds, Host tropism

## Abstract

Complement has been considered as an important factor impacting the host–pathogen association of spirochetes belonging to the *Borrelia burgdorferi* sensu lato complex, and may play a role in the spirochete’s ecology. Birds are known to be important hosts for ticks and in the maintenance of borreliae. Recent field surveys and laboratory transmission studies indicated that certain avian species act as reservoir hosts for different *Borrelia* species. Nevertheless, our current understanding of the molecular mechanisms determining host tropism of *Borrelia* is still in its fledgling stage. Concerning the role of complement in avian-host tropism, only a few bird species and *Borrelia* species have been analysed so far. Here, we performed in vitro serum bactericidal assays with serum samples collected from four bird species including the European robin *Erithacus rubecula*, the great tit *Parus major*, the Eurasian blackbird *Turdus merula*, and the racing pigeon *Columba livia*, as well as four *Borrelia* species (*B. afzelii*, *B. garinii*, *B. valaisiana*, and *B. burgdorferi* sensu stricto). From July to September 2019, juvenile wild birds were caught using mist nets in Portugal. Racing pigeons were sampled in a loft in October 2019. Independent of the bird species analysed, all *Borrelia* species displayed an intermediate serum-resistant or serum-resistant phenotype except for *B. afzelii* challenged with serum from blackbirds. This genospecies was efficiently killed by avian complement, suggesting that blackbirds served as dead-end hosts for *B. afzelii*. In summary, these findings suggest that complement contributes in the avian–spirochete–tick infection cycle and in *Borrelia*-host tropism.

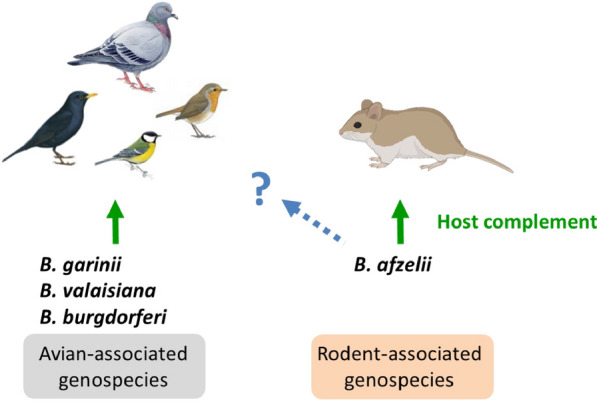

Like other blood-borne pathogens, spirochetes belonging to the *Borrelia burgdorferi* sensu lato (s.l.) complex circulate in nature in a complex vector-host transmission cycle without causing symptoms in their reservoir hosts [1]. To date, at least six *Borrelia* species have been unambiguously associated with human Lyme disease or borreliosis including *B. garinii*, *B. afzelii*, *B. burgdorferi* sensu stricto (s.s.), *B. spielmanii*, *B. bavariensis*, and *B. mayonii* [2]*.* Concerning the human pathogenic potential of *B. lusitaniae* and *B. valaisiana*, only a few cases have been reported so far [2]. Apparently, *Borrelia* species developed certain means to survive for prolonged times in their hosts in a “silent” or unrecognizable manner, like a Trojan horse, possibly leading to a long-lasting infection in their hosts that has a considerable impact on *Borrelia* ecology. Complement evasion is considered to be one of the driving factors determining the host reservoir competence for *Borrelia* [2–4]. In contrast to non-reservoir or dead-end hosts like humans and certain vertebrates (e.g., deer), reservoir hosts allow *Borrelia* to survive and multiply in their tissues and, more importantly, are capable of transmitting spirochetes to feeding ticks, and thus maintaining the bacteria in circulation [1].

Concerning avian species, reservoir competence has been experimentally proven in several transmission studies [5–10] as well as in field studies in which feeding larvae were collected from avian hosts [5, 11, 12]. Under the assumption that transovarial transmission of *B. burgdorferi* s.l. is very rare in ixodid ticks [13], infections in fed larvae collected from birds captured in the field have been used as a proxy of reservoir host competence. In addition, serum susceptibility may also provide information on potential vertebrate reservoir hosts. Studies aiming at determining serum susceptibility have been performed for several vertebrates including avian, reptilian, rodent, and ruminant hosts (reviewed in [2]). Interestingly, data gathered from serum susceptibility studies with pheasants (*Phasianus colchicus*), blackbird (*Turdus merula*), seabird (unknown species), and quail (*Coturnix* sp.) indicated that (i) *B. valaisiana* and *B. garinii* are mainly resistant to avian complement, (ii) *B. burgdorferi* s.s. displayed an intermediate serum resistance phenotype, and (iii) *B. afzelii*, *B. bavariensis*, and *B. lusitaniae* were largely susceptible to avian sera (reviewed in [2] and [3]). These findings revealed a strong correlation between the ability of a particular *Borrelia* species to resist complement-mediated killing and its capability to infect and survive in the host from where the serum originates. Thus, rodent-associated genospecies are killed by avian serum whereby avian-associated genospecies are highly susceptible to rodent serum. Moreover, Passeriformes and gallinaceous bird species appeared to be capable of serving as reservoirs for *B. garinii*, *B. valaisiana*, and *B. burgdorferi* s.s. (hereafter referred to as *B. burgdorferi*), but not for *B. afzelii*, *B. lusitaniae*, *B. japonica*, and *B. bavariensis.*

The specific infection pattern supports the notion that complement is a driving factor for host tropism of Lyme borreliae. To gain further insight into the complement-host association pattern, we performed serum-bactericidal assays with avian sera collected from juveniles of four bird species captured in Portugal: European robin *Erithacus rubecula* (hereafter robin), great tit *Parus major*, Eurasian blackbird *Turdus merula* (hereafter blackbird), and racing pigeon *Columba livia* and four *Borrelia* species (*B. afzelii*, *B. garinii*, *B. valaisiana*, and *B. burgdorferi*). Our pilot study also aimed to better understand the findings on the reservoir competence of avian species for Lyme borreliae [6, 7, 14], in particular for those *Borrelia* genospecies commonly found in Europe.

Wild robins (*n* = 14), great tits (*n* = 16), blackbirds (*n* = 11), and racing pigeons (*n* = 9) were captured with mist nets from July to September 2019 in Vale Soeiro (40°19′ N, 8°24′ W), Portugal. We sampled blackbird, great tit, and robin juvenile birds (hatched in the previous breeding season). The sampled birds were ringed and carefully inspected for feeding ticks. Racing pigeons were sampled from a loft in Antanhol (40°09′ N, 8°27′ W), Coimbra, Portugal. None of the birds were infested with feeding ticks at the time of capture. In addition, cattle and goat serum were included as controls.

Blood was collected from the brachial vein into tubes containing clot activator (BD Microtainer tubes, Becton Dickinson, Spain, catalogue number 365967). The blood was immediately placed in a cool box until transport to the laboratory where it was centrifuged for 10 min at 14,000×*g* and 4 °C within a maximum of 9 h from collection. The serum was transferred into a new vial and frozen immediately at −80 °C until analyses.

*Borrelia* strains *B. afzelii* FEM1 (skin isolate, Germany), *B. garinii* G1 (CSF, Germany), *B. valaisiana* VS116 (type strain, tick isolate, Switzerland), *B. burgdorferi* B31 (ATCC^*®*^ 35210, tick isolate, USA), and *B. lusitaniae* PoHL (skin isolate, Portugal) were cultured until mid-exponential phase (5 × 10^7^ spirochetes per ml) at 33 °C in modified Barbour-Stoenner-Kelly (BSK-H) medium (Bio & Sell GmbH, Feucht, Germany).

The *Borrelia* strains used in this study have been selected for the following reasons: (i) they represent the most frequently isolated genospecies in ixodid ticks in Europe [5, 7, 11, 15], and (ii) the susceptibility/resistance pattern to human serum did not change in repeated studies. For instance, *B. afzelii* FEM1 always displayed a serum-resistant phenotype, *B. garinii* G1, *B. valaisiana* VS116, and *B. lusitaniae* PoHL were strongly serum-susceptible, and *B. burgdorferi* B31 could be categorized as intermediate serum-resistant [2]. In addition, the motility, viability, and morphology of each strain was inspected by dark-field microscopy to be sure that only highly viable spirochetes were used in the experiments.

For screening of anti-*Borrelia* IgG in the collected bird samples, a highly sensitive ELISA was used (Anti-*Borrelia* Plus VlsE ELISA IgG, EUROIMMUN, Lübeck, Germany) containing whole cell extracts of *B. afzelii*, *B. garinii*, and *B. burgdorferi* as well as recombinant OspC and VlsE. The protocol was performed according to the manufacturer’s recommendation except that sera were diluted 1:20 prior testing. Calibrators, standards, and the serum samples were added to the wells, and microtiter plates were incubated for 30 min at room temperature. After washing, antigen–antibody complexes were detected after an incubation of 30 min by using horseradish peroxidase (HRP)-conjugated goat anti-bird IgG antibody (abcam, Cambridge, UK) (dilution 1:50,000). Afterwards, the ready-to-use substrate was added, and the reactions were terminated after an incubation period of 15 min. The absorbance was measured at 450 nm. The final interpretation of the test results are as follows: values < 0.8 were negative, values between ≥ 0.8 < 1.1 are borderline, and values ≥ 1.1 were considered positive.

Serum susceptibility of spirochetes to bird sera was assessed as previously described with slight modifications [16]. Briefly, spirochetes grown at mid-logarithmic phase were sedimented by centrifugation, resuspended in 500 µl BSK medium, and counted by dark-field microscopy. Reaction mixtures consisting of 20 µl of highly viable spirochetes (4 × 10^7^ cells per ml) and 20 µl of avian serum were incubated at 38 °C with gentle agitation (350 rpm). The percentage of viable spirochetes was determined by dark-field microscopy after 0, 3, and 6 h. Spirochetes in nine microscopy fields were counted for each time point per strain and serum sample using Glasstic slides 10 (KOVA International Inc., CA, USA). Due to the small amount of blood collected from the individual bird, each *Borrelia* species was tested against serum samples of three different individuals of each bird species (except blackbird, *n* = 2). Of note, five out of seven serum samples collected from blackbirds showed haemolysis which leads to an agglutination of spirochetes immediately after adding to the reaction mixture (data not shown). The respective serum resistance pattern was defined according to the spirochetes’ motility and classified as follows: serum-resistant phenotype, 75–100% of viable spirochetes; intermediate serum-resistant phenotype, 30–75% of viable spirochetes; serum-sensitive phenotype, 0–30% of viable spirochetes [17]. The percentages of viable spirochetes were calculated by setting the number of motile bacteria at 0 h of incubation to 100%. Data were visualized by using GraphPad Prism version 7.04. Means are presented with standard deviation (SD).

To determine complement activity of the collected serum samples, the reptile-associated genospecies *B. lusitaniae* was employed. Due to the limited number of bird sera, only a single serum sample from *P. major* was analysed. As further controls, all *Borrelia* strains investigated were also exposed to cattle and goat sera, and viable spirochetes were counted after 3 and 6 h as described above.

To further assess to role of complement as a factor involved in avian-host association of Lyme borreliae, we investigated blood samples from juvenile birds, including two individual blackbirds, 12 great tits, 12 robins, and six racing pigeons. In addition, all serum samples investigated were screened for the presence of anti-*Borrelia* IgG antibodies. None of the serum samples tested were anti-*Borrelia*-positive except two serum samples collected from blackbirds, both of which were considered borderline (0.83 and 0.99, respectively).

Serum susceptibility testing was conducted by counting viable spirochetes by dark-field microscopy after an incubation period of 0, 3, and 6 h, as previously described [16]. As demonstrated in Fig. 1a, *B. garinii* G1, *B. valaisiana* VS116, and *B. burgdorferi* B31 displayed a resistant or an intermediate serum-resistant phenotype to all bird species investigated (mean percentage of viable spirochetes after 6 h of incubation: 75 ± 15%, range = 42–97%). The data show that *B. afzelii* FEM1 resists complement-mediated killing when individual serum samples from great tits and robins were employed (mean percentage of motile spirochetes after 6 h of incubation with great tit and robin sera: 84 ± 13%). By contrast, when *B. afzelii* FEM1 was exposed to serum samples of blackbirds, an average of 66 ± 7% spirochetes had been killed after 3 h of incubation, and only 16 ± 9.6% of spirochetes survived serum treatment after 6 h (Fig. 1b).

**Fig. 1:**
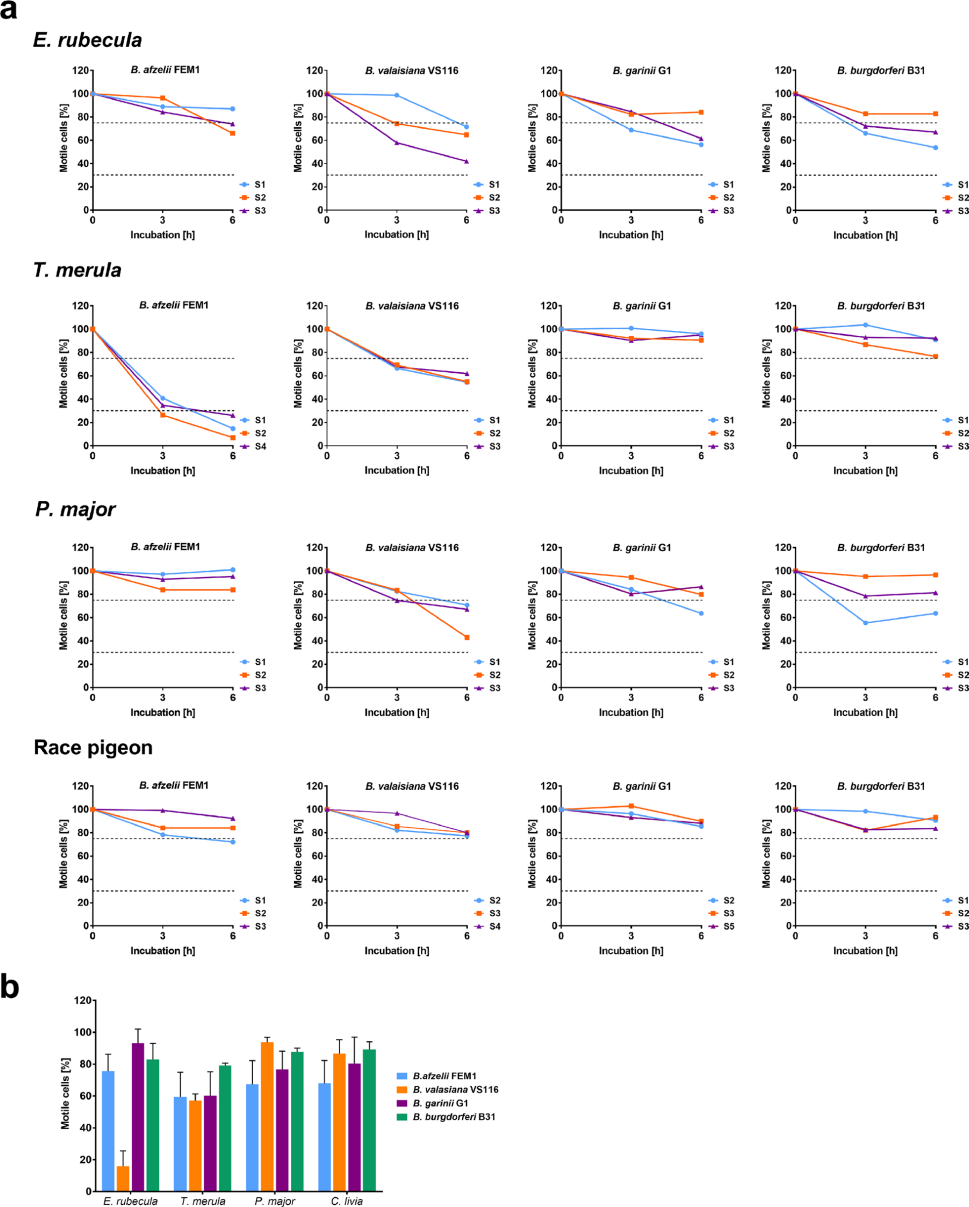
Serum susceptibility of Lyme borreliae exposed to bird serum samples. **a** Each genospecies and the respective avian species was independently challenged with serum samples from three individual birds, except for the sera of Eurasian blackbirds (*Turdus merula*, *n* = 2). **b** Percentage of viable spirochetes after 6 h of incubation with avian sera. The data represents three independent experiments for each *Borrelia* genospecies/bird species combination

Of note, all four *Borrelia* species survived in the presence of serum collected from racing pigeons (85 ± 6.5% of motile spirochetes after 6 h of incubation). The highest survival rates after 6 h of incubation was observed for *B. garinii* G1 treated with blackbird serum (94 ± 2.9%) and *B. afzelii* FEM1 incubated with serum of the great tit (93 ± 8.8%) (Fig. 1b). Employing dark-field microscopy, only 6.5% of the cells of *B. lusitaniae* PoHL survived, while 94% of the cells of *B. burgdorferi* B31 resist complement-mediated killing after 6 h of incubation in the presence of great tit serum (data not shown). This finding indicates that avian complement was still active after blood collection and long-term storage. Furthermore, all *Borrelia* strains challenged with cattle and goat serum were efficiently killed after 6 h of incubation (Fig. 2) as previously described [18, 19].

**Fig. 2:**
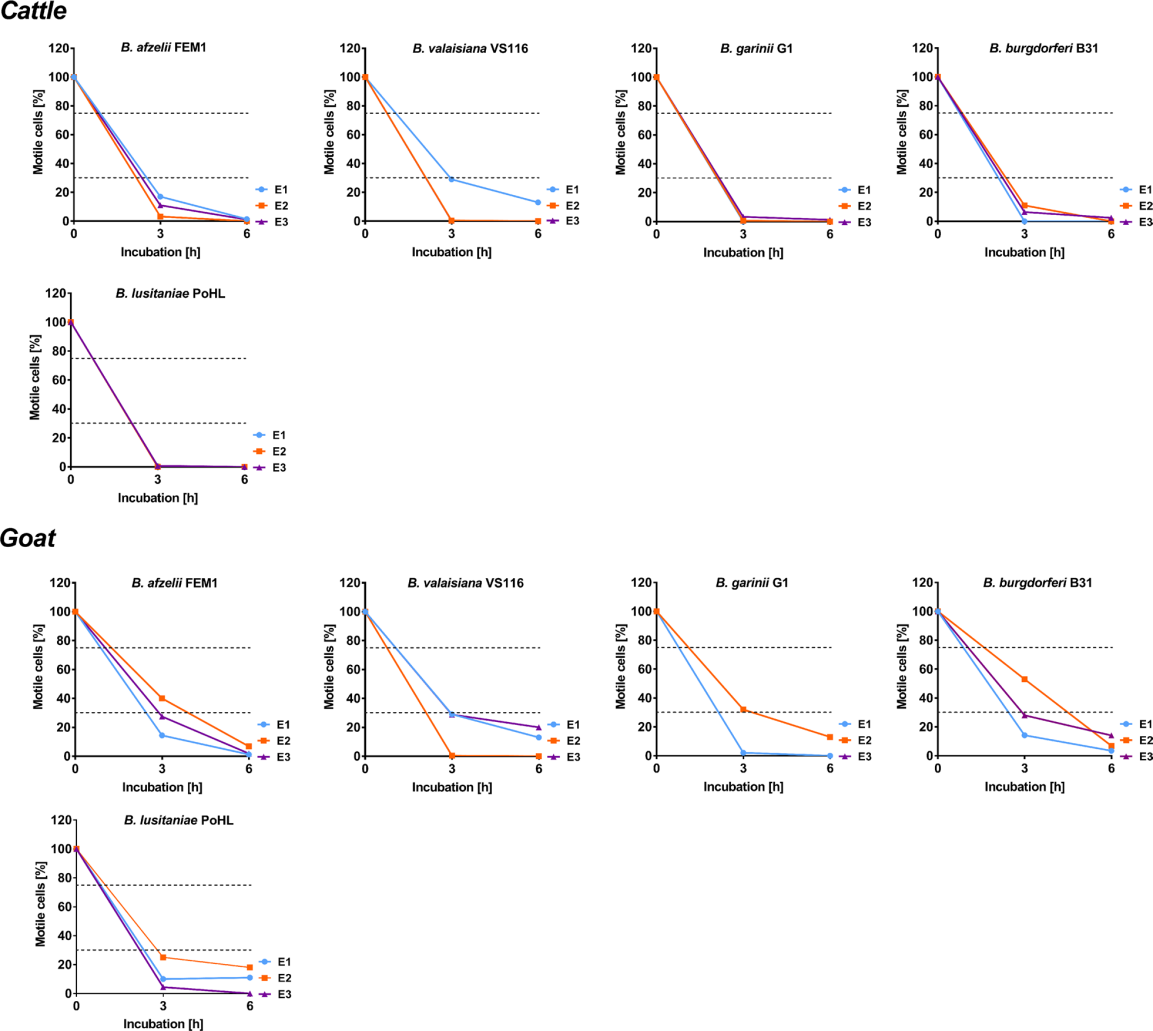
Bactericidal effect of control sera on the viability of Lyme borreliae. Each *Borrelia* species was treated with serum samples collected from cattle (*n* = 1) and goat (*n* = 1). Three independent technical replicates (E1 to E3) were performed

Emphasizing the potential impact of complement as an important factor determining host tropism of Lyme borreliae, here we analysed the serum susceptibility of the four main *Borrelia* genospecies in Europe to sera collected from juvenile wild birds. For comparison, and apart from blackbirds [3], we included great tits and robins, two wild ubiquitous bird species often infested with ixodid ticks [11, 20], and the racing pigeon as potential host for Lyme borreliae. The comparative analyses of our pilot study largely reiterate prior findings that avian-associated *Borrelia* species such as *B. garinii* and *B. valaisiana* resist complement-mediated killing to all serum samples analysed [3, 4, 21]. *B. burgdorferi* has previously been shown to resist complement-mediated killing by avian serum collected from pheasants and quail, both of which are utilized as model organisms for elucidating host association of Lyme borreliae [8, 21, 22, 23]. As expected, growth of *B. afzelii* FEM1 was strongly affected by blackbird serum as also demonstrated in previous studies in which pheasants (*Phasianus colchicus*), blackbirds, and a seabird were examined [3]. In contrast to previous findings, our data revealed for the first time that *B. afzelii* appears to survive in the presence of serum of robins, great tits, and racing pigeons, suggesting a species-specific host association/tropism of certain *Borrelia* species as discussed more recently for *B. burgdorferi* and *B. bavariensis* [2, 24].

Although numerous field studies and transmission experiments suggested that birds including great tits and robins play a limited or even a negligible role in maintaining *B. afzelii* in circulation [5, 6, 25], previous findings of *B. afzelii*-infected larvae and nymphs that fed on birds revealed that certain bird species, including great tits [11, 26], robins [14], dunnocks, and song thrushes [6], might serve as potential reservoir hosts or transmitters for certain *B. afzelii* strains and variants, respectively [14, 15]. *B. afzelii*-infected larvae could result from co-feeding or incomplete blood meal on another infected vertebrate host [15]. In addition, Heylen et al. [6] found that pathogen-free larval *Ixodes ricinus* ticks co-feeding with *B. afzelii*-infected *I. ricinus* nymphal ticks on naive blackbirds did not become infected with *B. afzelii*, but a low percentage of infected larvae were detected when this experiment was performed in naïve great tits. These findings suggest that avian blood of naïve great tits and blackbirds was bactericidal to particular *B. afzelii* strains or variants and, thus, provide additional evidence for a strain-specific host association. It is tempting to speculate that the concept of a restricted genospecies-specific host association of Lyme borreliae (i.e., division into rodent-associated, avian-associated, and reptile-associated genospecies) has to be readjusted, in particular due to recent observations revealing more diverse relationships of Lyme borreliae with their hosts in nature as previously thought.

Concerning *B. garinii* and *B. valaisiana*, both genospecies have predominately been identified in ticks collected from ground-dwelling and migrating songbirds such as tree pipits, blackbirds, great tits, robins, and song thrushes, revealing a strong association of these *Borrelia* genospecies with distinct avian hosts [15]. In line with these findings, we showed that *B. garinii* and *B. valaisiana* exhibited an intermediate serum-resistant or serum-resistant phenotype to the sera of all four wild birds tested in our study (Fig. 1).

In contrast to the United States where *B. burgdorferi* is the main *Borrelia* species causing Lyme disease [27, 28, 30, 31], the competence of birds as reservoir hosts has not fully been elucidated in Europe [29]. Here, we showed that *B. burgdorferi* displayed either an intermediate serum-resistant (robin) or a serum-resistant (blackbird, great tit, and racing pigeon) phenotype to avian sera (Fig. 1). These findings are also in close agreement to the data obtained from previous serum bactericidal assays demonstrating that *B. burgdorferi* was partially resistant to blackbird, pheasant, and seabird sera [3, 4]. In summary, our data suggest that *Borrelia*-specific factors along with the avian innate immune system contributes to host speciation and competence as proposed recently [2].

The racing pigeon is thought to have a negligible or even a minor role in the *B. burgdorferi* s.l. transmission cycle, as those birds are largely maintained in captivity. Nevertheless, free-living pigeons are known to carry several zoonotic pathogens [32] and, thus, may contribute to the circulation of certain pathogens. Further investigations aiming at elucidating the reservoir competence of free-living pigeons will undoubtedly complement our findings indicating that serum of pigeons did not exhibit a borreliacidal effect on spirochetes; thus a potential role of this bird species in *B. burgdorferi* s.l. transmission cannot be completely discounted.

All serum samples analysed derived from birds that were captured in the wild, except for the sera collected from racing pigeons. Although none of the birds were infested with ticks at the time of capturing, we cannot completely rule out that they have had previous contact with *Borrelia*-infected ticks. However, those were juvenile birds, born in the previous spring, and the tick activity in this region is relatively low during warm and dry summer months [20], reducing the probability of contact of these birds with questing ticks. In addition, serological tests confirmed that none of the sera investigated were positive for anti-*Borrelia* IgG antibodies except the two samples collected from blackbirds showing a borderline result. Of note, *B. afzelii* FEM1 also displayed a serum-sensitive phenotype when an anti-*Borrelia*-negative blackbird sample was used for the bactericidal assays (data not shown). This finding suggested that *B. afzelii* FEM1 was primarily killed by the activation of the alternative pathway, but not through an antibody-dependent complement activation via the classical pathway. Also, the data of the serum bactericidal assays presented herein revealed a high consistency when different individual sera were tested with a given *Borrelia* species, suggesting that those birds were likely naïve.

Although serum complement is a limiting factor affecting *Borrelia* survival in the tick and directly after transmission to the vertebrate host, the inferences on vertebrate reservoir competence should take into consideration additional factors such as the heterogeneity of proteins interacting with the host immune system among individual *Borrelia* strains as well as host-specific factors like the physiological state and age of the birds that also may affect the host’s competence. Moreover, genetic polymorphism among avian host populations cannot be excluded because co-evolution between host and spirochetes might also contribute to a strain-specific serum susceptibility pattern. In summary, the data presented herein not only confirm previous findings but add new data on the complexity of a complement-driven host tropism among *Borrelia* in nature*.*

## Data Availability

The datasets generated during and/or analysed during the current study are available from the corresponding author on reasonable request.

## References

[1] Radolf JD, Caimano MJ, Stevenson B, Hu LT (2012). Of ticks, mice and men: understanding the dual-host lifestyle of Lyme disease spirochaetes. Nat Rev Microbiol.

[2] Lin YP, Diuk-Wasser MA, Stevenson B, Kraiczy P (2020). Complement evasion contributes to Lyme borreliae-host associations. Trends Parasitol.

[3] Kurtenbach K, De Michelis S, Etti S, Schafer SM, Sewell HS, Brade V (2002). Host association of *Borrelia burgdorferi* sensu lato-the key role of host complement. Trends Microbiol.

[4] Kurtenbach K, Sewell H-S, Ogden NH, Randolph SE, Nuttall PA (1998). Serum complement sensitivity as a key factor in Lyme disease ecology. Infect Immun.

[5] Heylen D, Matthysen E, Fonville M, Sprong H (2014). Songbirds as general transmitters but selective amplifiers of *Borrelia burgdorferi* sensu lato genotypes in *Ixodes rinicus* ticks. Environ Microbiol.

[6] Heylen DJ, Sprong H, Krawczyk A, Van Houtte N, Genne D, Gomez-Chamorro A (2017). Inefficient co-feeding transmission of *Borrelia afzelii* in two common European songbirds. Sci Rep.

[7] Humair PF, Postic D, Wallich R, Gern L (1998). An avian reservoir (*Turdus merula*) of the Lyme borreliosis spirochetes. Zentralbl Bakteriol.

[8] Kurtenbach K, Carey D, Hoodless AN, Nuttall PA, Randolph SE (1998). Competence of pheasants as reservoirs for Lyme disease spirochetes. J Med Entomol.

[9] Norte AC, de Carvalho IL, Nuncio MS, Ramos JA, Gern L (2013). Blackbirds *Turdus merula* as competent reservoirs for *Borrelia turdi* and *Borrelia valaisiana* in Portugal: evidence from a xenodiagnostic experiment. Environ Microbiol Rep.

[10] Richter D, Spielman A, Komar N, Matuschka FR (2000). Competence of American robins as reservoir hosts for Lyme disease spirochetes. Emerg Infect Dis.

[11] Heylen D, Tijsse E, Fonville M, Matthysen E, Sprong H (2013). Transmission dynamics of *Borrelia burgdorferi* s.l. in a bird tick community. Environ Microbiol.

[12] Norte AC, Ramos JA, Gern L, Nuncio MS, de Carvalho IL (2013). Birds as reservoirs for *Borrelia burgdorferi* s.l. in western Europe: circulation of *B. turdi* and other genospecies in bird-tick cycles in Portugal. Environ Microbiol.

[13] van Duijvendijk G, Coipan C, Wagemakers A, Fonville M, Ersoz J, Oei A (2016). Larvae of *Ixodes ricinus* transmit *Borrelia afzelii* and *B. miyamotoi* to vertebrate hosts. Parasit Vectors.

[14] Franke J, Moldenhauer A, Hildebrandt A, Dorn W (2010). Are birds reservoir hosts for *Borrelia afzelii*?. Ticks Tick Borne Dis.

[15] Marie-Angele P, Lommano E, Humair PF, Douet V, Rais O, Schaad M (2006). Prevalence of *Borrelia burgdorferi* sensu lato in ticks collected from migratory birds in Switzerland. Appl Environ Microbiol.

[16] Walter L, Sürth V, Röttgerding F, Zipfel PF, Fritz-Wolf K, Kraiczy P (2019). Elucidating the immune evasion mechanisms of *Borrelia mayonii*, the causative agent of Lyme disease. Front Immunol.

[17] Kraiczy P, Hunfeld KP, Breitner-Ruddock S, Wurzner R, Acker G, Brade V (2000). Comparison of two laboratory methods for the determination of serum resistance in *Borrelia burgdorferi* isolates. Immunobiology.

[18] Kraiczy P (2016). Travelling between two worlds: complement as a gatekeeper for an expanded host range of Lyme disease spirochetes. Vet Sci.

[19] Bhide MR, Travnicek M, Levkutova M, Curlik J, Revajova V, Levkut M (2005). Sensitivity of *Borrelia* genospecies to serum complement from different animals and human: a host-pathogen relationship. FEMS Immunol Med Microbiol.

[20] Norte AC, de Carvalho IL, Ramos JA, Goncalves M, Gern L, Nuncio MS (2012). Diversity and seasonal patterns of ticks parasitizing wild birds in western Portugal. Exp Appl Acarol.

[21] Frye AM, Hart TM, Tufts DM, Ram S, Diuk-Wasser MA, Kraiczy P (2020). A soft tick *Ornithodoros moubata* salivary protein OmCI is a potent inhibitor to prevent avian complement activation. Ticks Tick Borne Dis.

[22] Kurtenbach K, Peacey M, Rijpkema SG, Hoodless AN, Nuttall PA, Randolph SE (1998). Differential transmission of the genospecies of *Borrelia burgdorferi* sensu lato by game birds and small rodents in England. Appl Environ Microbiol.

[23] Kurtenbach K, Schafer SM, Sewell HS, Peacey M, Hoodless A, Nuttall PA (2002). Differential survival of Lyme borreliosis spirochetes in ticks that feed on birds. Infect Immun.

[24] Margos G, Fingerle V, Reynolds S (2019). *Borrelia bavariensis*: vector switch, niche invasion, and geographical spread of a tick-borne bacterial parasite. Front Ecol Evol.

[25] Norte AC, Margos G, Becker NS, Albino Ramos J, Nuncio MS, Fingerle V (2020). Host dispersal shapes the population structure of a tick-borne bacterial pathogen. Mol Ecol.

[26] Dubska L, Literak I, Kocianova E, Taragelova V, Sychra O (2009). Differential role of passerine birds in distribution of *Borrelia spirochetes*, based on data from ticks collected from birds during the postbreeding migration period in central Europe. Appl Environ Microbiol.

[27] Brinkerhoff RJ, Bent SJ, Folsom-O'Keefe CM, Tsao K, Hoen AG, Barbour AG (2010). Genotypic diversity of *Borrelia burgdorferi* strains detected in *Ixodes scapularis* larvae collected from North American songbirds. Appl Environ Microbiol.

[28] Ginsberg HS, Buckley PA, Balmforth MG, Zhioua E, Mitra S, Buckley FG (2005). Reservoir competence of native North American birds for the lyme disease spirochete *Borrelia burgdorferi*. J Med Entomol.

[29] Heylen D, Krawczyk A, de Carvalho IL, Nuncio MS, Sprong H, Norte AC (2017). Bridging of cryptic *Borrelia* cycles in European songbirds. Environ Microbiol.

[30] Newman EA, Eisen L, Eisen RJ, Fedorova N, Hasty JM, Vaughn C (2015). *Borrelia burgdorferi* sensu lato spirochetes in wild birds in northwestern California: associations with ecological factors, bird behavior and tick infestation. PLoS ONE.

[31] Scott JD, Anderson JF, Durden LA (2012). Widespread dispersal of *Borrelia burgdorferi*-infected ticks collected from songbirds across Canada. J Parasitol.

[32] Haag-Wackernagel D, Moch H (2004). Health hazards posed by feral pigeons. J Infect.

